# Differential socioemotional and educational profiles in early childhood and primary education students in a lockdown situation

**DOI:** 10.3389/fpsyg.2024.1296333

**Published:** 2024-05-09

**Authors:** Noemí Serrano-Díaz, Estíbaliz Aragón, Rosario Merida-Serrano

**Affiliations:** ^1^Department of Didactic, Faculty Science of Education, University of Cadiz, Puerto Real, Spain; ^2^Department of Psychology, Faculty Science of Education, University of Cadiz, Puerto Real, Spain; ^3^Department of Didactic, Faculty Science of Education, University of Cordoba, Cordoba, Spain

**Keywords:** child development, informal learning, family, socialization, lockdown

## Abstract

Recent lockdown situations have highlighted social relationship deprivation of schoolchildren and the need to develop the teaching-learning process in informal contexts. The aim of this study was to analyze differential socioemotional and educational profiles in Early Childhood and Primary Education students using variables relating to academic performance during the COVID-19 pandemic. Correlational, descriptive, and inferential statistical analyses were performed that yielded differential explanatory models depending on the stage of education. The results reveal statistically significant differences in all the variables except for family-school relationship. In the linear regression models, the most statistically significant variable for school performance in both stages was family-school relationship. However, differences were found between both profiles: emotional impact for Early Childhood Education students and social impact for Primary Education students. Lastly, leisure activities at home were included as an explanatory variable only in the Primary Education regression model. The final conclusions highlight the need to attend to the evolutionary characteristics of students in each stage to improve school performance in similar lockdown situations.

## 1 Introduction

In theory and in practice, student education involves much more than acquiring conceptual learning, given that teaching should also provide developing children with comprehensive and holistic education. In essence, the aim, along with conceptual learning, should be to transmit other practical “to do” learning as well as values, i.e., teaching how “to be” (or attitudinal content) (Case, 2020; Ponomariovienė and Jakavonytė-Staškuvienė, 2022). The aim behind such education is to build a knowledge base for a changing society to instigate transformation of social reality (Batista et al., 2017).

Many authors agree that it is important to focus on emotional education for future generations: an aspect that is essential for social education in the early stages of human development (Petrides et al., 2018; Alwaely et al., 2021; Martí et al., 2022). Therefore, it is imperative that children cultivate relationships with their peers, and learn how to recognize, monitor and regulate their emotions. They also need to establish pro-social behaviors and behavioral patterns that are effective in future adult relationships (Bisquerra-Alzina, 2019; Flook et al., 2019). Social interactions between peers were transformed by the COVID-19 pandemic leading to a drastic change in lifestyle that generated consequences at both a social and an emotional level. In Spain, the state of emergency declared in March 2020 (Government Spanish, 2020) meant that people were confined to their homes to prevent the spread of the virus (Siqueira et al., 2020). This culminated in a series of consequences for schoolchildren, their teachers, their education and their families (Chandra, 2020). Interruption to face-to-face educational activities had a socioemotional impact on children due to social distancing and peer relationship deprivation (Álvarez-Zarzuelo, 2020; Balluerka-Lasa et al., 2020; Chandra, 2020; Sandín et al., 2021). School activities took place at home, where they were combined with leisure activities. Studies on the impact of the COVID-19 situation on academic performance have recently been published which reveal that at least half an academic term has been lost, specifically in mathematical education in public schools (Arenas et al., 2022). Teachers were forced to adapt telematics and distance learning from one day to the next, which highlighted the need for training in the use of technology in EDTECH education (Sánchez-Mendiola et al., 2020; Sandín et al., 2021). Families were also faced with the need to make huge efforts. Parents had to take on new roles as teachers, as well as establishing a closer relationship with schools, albeit online, which highlighted the gap (digital divide) in terms of socioeconomic inequalities (Lloyd, 2020). This underlined the need for more frequent and efficient communication, demonstrating, once again, the importance of the family-school relationship in schoolchildren’s performance.

School success depends largely on family support. This dependency was increased by the confinement situation during the COVID-19 pandemic. Furthermore, according to Sánchez and Dávila (2022), parents’ expectations are directly related to the support they provide to their children; consequently, the confinement situation facilitated interactions between parents and children, increasing their participation and, therefore, their satisfaction and expectations toward school performance (Manzano-León et al., 2022; Somogyi et al., 2023). All of this confirms the determining role of parental involvement in school performance during the pandemic (Serrano-Díaz et al., 2022).

The exceptional situation suffered by society led to an interest in determining how the teaching-learning process was approached by families and the impact of COVID-19 on education at different stages of development in Early Childhood and Primary Education school children.

Due to students’ particularities and different levels of maturity, both stages of education differ from each other in their methodological principles. The great psychology theorists of the 20th century highlight that social relationships, initially with adults and later with peers, are evident in children’s formation and development. Specifically, students learn in society not only by trial and error but by imitation owing to the activation of so-called “mirror neurons”. Thus, children’s learning comes from interaction with their physical and social environment (Piaget and Inhelder, 1973; Vigotsky, 1979; Bandura, 1986).

Consequently, Early Childhood Education students aged 3–6 are at a stage where family and parents exert the greatest influence, which highlights the need for adult role models (Feldman and Bishop, 2005; Calmaestra et al., 2018). In contrast, the development of Primary Education students places greater emphasis on their dependence on peers, who provide them with emotional support. The emotional and social needs for effective development vary between both stages. On the one hand, children in Early Childhood Education are in a stage of basic learning that requires a close relationship with reference adults. On the other hand, primary school children need greater openness to other learning contexts (Feldman and Bishop, 2005; Páramo, 2008; James, 2021). Therefore, the school is considered a social institution that trains people and offers the opportunity to establish interpersonal relationships and bonds beyond the family context (Jara-Parra and Jara-Parra, 2020; Ravens-Sieberer et al., 2022).

On the other hand, play is also an important activity for children’s physical and psychological wellbeing. The game is considered a learning vehicle since it favors development in the different areas of the educational curriculum. Furthermore, it can be considered as an instrument for the comprehensive development of the child. Therefore, we can distinguish between play with pedagogical intention induced by the teacher and free game that is necessary in both stages and that contributes to the construction of the individual’s identity (Taylor and Boyer, 2020; Kvintova et al., 2022).

Time dedicated to play and leisure is fundamental at such young ages, even more so in periods of lockdown where schoolwork and leisure often concur in the same space. A challenging situation for both activities with the latter trying to fulfil its role as a work break and much needed resting period needed to recharge energy levels (Gallardo-López and Gallardo-Vázquez, 2018; Varela et al., 2021). For many authors, the importance of play as an educational tool is paramount owing to its many benefits to the integral development of the person (Gallardo-López and Gallardo-Vázquez, 2018; Varela et al., 2021).

The aim of this study was to perform an in-depth analysis of the impact of lockdowns on students in Early Childhood and Primary Education in order to compare both groups and analyze differential aspects that could contribute to better intervention strategies in future situations similar to the COVID-19 pandemic.

To this end, the following objectives were set:

*O1*. Analyze the differences between Early Childhood and Primary Education students using variables considered relevant for coping with academic performance during periods of lockdown (social impact, emotional impact, leisure activities, co-responsibility and family-school relationship).

*O2*. Analyze the correlations between the set of variables relating to education in situations of lockdown in Early Childhood and Primary Education students.

*O3*. Determine differential explanatory models based on the stage of education that reveal the contribution of the predictor variables to variability in academic performance.

The study is based on three hypotheses:

*H1*. The predictor variables (social impact, emotional impact, leisure activities, co-responsibility and family-school relationship) will show significant differences in favor of Early Childhood Education students that will explain better coping mechanisms for their academic performance in lockdown.

*H2*. The variables analyzed (social impact, emotional impact, leisure activities, co-responsibility, family-school relationship, academic performance at home) will correlate for both Early Childhood and Primary Education students.

*H3*. Differential explanatory models will reveal the contribution of predictor variables to variability in academic performance depending on the stage of education and its intrinsic characteristics.

## 2 Materials and methods

### 2.1 Participants

The participants in this study were adults distributed by gender given that women more than men are generally more involved in the supervision and care of their children, which increases the burden of family responsibilities that make work-life balance difficult (Álvarez et al., 2019). There were 529 participants aged 20–63 (Mage = 39.94 SD = 6.33). A total of 64 participants were male (Mage = 43.58; SD = 6.15) compared to 465 who were female (Mage = 39.44; SD = 6.02).

Of the total number of schoolchildren whose relatives were surveyed, 25.5% were in Early Childhood Education (*n* = 135) and 74.5% (*n* = 394) Primary Education. In Spain, where the study is carried out, the Early Childhood Education stage is divided into two substages of 0–3 years and 3–6 years, specifically this study is carried out in the 3–6 years stage. On the other hand, Primary Education covers the period from 6 to 12 years. The perceptions of the parents of students in Primary Education correspond to the three stages of formal primary education in Spain. Therefore, the groups are proportional in accordance with the educational stage. Consequently, the students considered in the study range from 3 to 12 years.

### 2.2 Instruments

An *ad-hoc* questionnaire, the Childhood Educational Impact Questionnaire Impact of COVID-19 on Education (CIEN) was used. The CIEN questionnaire is the result of a pilot study performed with 400 participants, which was validated by peer review (Pedrosa et al., 2013). After a factor analysis, a final questionnaire was obtained consisting of 63 items grouped into 6 dimensions:

- Co-responsibility: understood as the sharing of family commitments, responsibilities and chores relating to school and leisure-time, as well as domestic and care issues.
- Emotional impact: refers to participants’ perceptions of their children’s feelings of joy, sadness, fear, anger, nervousness, etc.
- Social impact: the participants’ perception of their children’s attitudes to feelings of loneliness, boredom, situations of family tension and nostalgia for school, friends and non-cohabiting family members.
- Impact on activities at home: understood as hours spent on supervised leisure activities (family play, reading, collaboration in household chores, etc.) and digital devices, both alone and supervised.
- Impact on school activities (performed at home): the perception of learning and its development under the guidance of teachers (quality and relevant use of online tools, appropriate homework assignments and motivation).
- Impact on the family-school relationship: refers to the communication between families, teachers and students. This dimension also addresses the monitoring of online class attendance, the learning pace and the perception of accompaniment.

The reliability of the questionnaire was calculated using Cronbach’s alpha which gave α = 0.81.

The questionnaire was given individually to parents with the aim of determining their perception on the socioemotional state of their children, the relationship between family and school, and the type of both leisure and school activities.

### 2.3 Data analysis

In order to perform the study on differences between Early Childhood and Primary Education students in a lockdown situation and the psychoeducational variables involved, descriptive and inferential statistical analyses were performed using the SPSS version 22 statistical package (descriptive statistics, correlations, Mann–Whitney U test and effect size). Linear regression analyses were also performed to study the models that best explained the variability in school children’s performance according to parents’ perceptions. There were no missing cases in the study given and the data was collected at a single point in time and an *ex post facto* design was used.

## 3 Results

The results were obtained from correlational, descriptive and inferential statistical analyses of the educational stages studied.

Table 1 shows that there were significant differences between the Early Childhood and Primary Education stages in the different variables analyzed, except in family-school relationship.

**Table 1:** Descriptive statistics, results of the inferential Mann–Whitney U test and effect size for the early childhood and primary education groups for the different variables analyzed.

	Childhood education	Primary education			
	*M (DT)*	*M (DT)*	*Sig.*	*d*	*R*
Co-responsibility	24.35 (5.27)	23.25 (5.5)	0.034**	0.20	0.10
Emotional Impact (negative)	14.79 (5.17)	15.94 (5.33)	0.022**	−0.21	−0.10
Social Impact (negative)	20.71 (4.52)	21.88 (4.75)	0.021**	−0.25	−0.12
Leisure activities at home	30.88 (3.89)	28.75 (4.39)	0.001**	0.51	0.24
Family-school Relationship	26.76 (6.59)	27.29 (6.84)	0.820	−0.07	−0.03
Educational activities and performance at home	39.66 (6.70)	38.05 (7.65)	0.030**	0.22	0.11

Similarly, the correlations between the different variables were calculated according to the stage of education.

Table 2 shows the correlations between the different variables studied in Early Childhood Education. It can be seen that the most significant correlation was obtained between parents’ perception of the family-school relationship and academic performance at home (*r* = 0.510). Similarly, a significant correlation was also observed between social and emotional impact (*r* = 0.426) followed by the correlation between negative emotional impact and academic performance at home (*r* = 0.385). Lastly, less significant correlations were obtained between leisure activities at home and family-school relationship (*r* = 0.285), and between the latter and co-responsibility (*r* = 0.255).

**Table 2:** Pearson’s bivariate correlations for the different variables relating to the impact of COVID-19 lockdowns on early childhood education.

	Co-responsibility	Emotional impact (negative)	Social impact (negative)	Leisure activities at home	Family-school relationship	Educational activities and performance at home
Co-responsibility	1	−0.053	−0.156	0.167	0.255**	0.128
Emotional impact (negative)	−0.053	1	426**	0.076	−0.125	−0.385**
Social impact (negative)	−0.156	0.426**	1	−0.020	−0.080	−0.134	Leisure activities at home	0.167	0.076	−0.020	1	0.285**	0.101
Family-school relationship	0.255**	−0.125	−0.080	0.285**	1	0.510**
Educational activities and performance at home	0.128	−0.385**	−0.134	0.101	0.510**	1

*p > 0.01, **p > 0.05.

In turn, Table 3 shows the values of the correlations between the variables analyzed at the Primary Education stage. As with the Early Childhood Education stage, the most significant correlation was obtained between parents’ perception of the family-school relationship and academic performance at home (*r* = 0.592). This was also followed by the correlation between the social and emotional impact (*r* = 0.526). To a lesser extent, significant correlations were found between academic performance at home and leisure activities (*r* = 0.293), leisure activities and family-school relationship (*r* = 0.267), social impact and academic performance at home (*r* = 0.262) as well as emotional impact and academic performance at home (*r* = 0.229). More correlations were found between the variables for the Primary Education stage than those for the Early Childhood Education stage, but those with more significant values were similar in both groups.

**Table 3:** Pearson’s bivariate correlations for the different variables relating to the impact of COVID-19 lockdowns on primary education.

	1. Co-responsibility	2. Emotional impact (negative)	3. Social impact	4. Leisure activities at home	5. Family-school relationship	6. Educational activities and performance at home
1. Co-responsibility	1					
2. Emotional Impact (negative)	−0.080	1				
3. Social Impact (negative)	−0.114	0.526**	1			
4. Leisure activities at home	0.119	−001	−0.128*	1		
5. Family-school Relationship	0.160**	−0.183**	−0.155**	−0.267**	1	
6. Educational activities and performance at home	0.126*	−0.229**	−0.262**	−293**	0.592**	1

*p > 0.01, **p > 0.05.

In order to contrast whether parents’ perception of academic performance at home varied according to school stage, a stepwise regression analysis was performed. The dependent variable was parents’ perception of academic performance at home, and the independent variables were co-responsibility, emotional impact, social impact, leisure activities at home and family-school relationship. Two different models were generated depending on the children’s stage at school.

The results of the linear regression analysis showed differentiated cognitive profiles depending on the stage of education. First, we present the results obtained in the stepwise regression analysis performed for the Early Childhood Education stage (Table 4). This analysis was performed in order to determine the significance of the different variables in the explanation of the dependent variable, in other words, in the perception of academic performance at home.

**Table 4:** Results of the stepwise linear regression analysis for the early childhood education stage.

				Change statistics				
Model	*R*	*R* ^2^	Adjusted *R*^2^	SE of the estimate	*R*^2^ change	*F* change	Sig. *F* change	Durbin Watson
1	0.507a	0.257	0.251	5.80	0.257	41.12	0.000	
2	0.604b	0.365	0.355	5.38	0.108	20.16	0.000	1.861

a. Predictors Variables: (Constant), Family-school relationship; b. Predictors Variables: (Constant), Family-school relationship; Emotional Impact (negative); c. Dependent Variable: Educational activities and performance at home.

Two models emerged from the regression analysis, with one offering greater explanatory power than the other. For Early Childhood Education, the multiple correlation coefficient was *R* = 0.604 and the coefficient of determination *R*^2^ = 0.365 which was adjusted to *R*^2^ = 0.355. The Durbin-Watson D statistic was calculated to determine the validity of the model giving a value of *D* = 1.86, which confirmed the absence of positive (values close to 0) and negative (values close to 4) autocorrelation. The dependent variable that better explained the variation was family-school relationship (β = 0.463) followed by emotional impact (β = −0.332) (Table 5).

**Table 5:** Coefficients of the proposed models for early childhood and primary education.

		B coefficient	Typ. error	β coefficient	Sig.
Early childhood education	Family-school relationship	0.471	0.075	0.463	0.000
	Emotional Impact	−0.430	0.096	−0.332	0.000
Primary education	Family-school relationship	0.608	0.048	0.544	0.000
	Social impact	−0.236	0.068	−0.141	0.001
	Leisure activities at home	0.241	0.074	0.139	0.001

In turn, an attempt was made to determine which variables explained the variability of academic performance at home in Primary Education students (Table 6).

**Table 6:** Results of the stepwise linear regression analysis for the primary education group.

				Change statistics				
Model	*R*	*R* ^2^	Adjusted R2	SE of the estimate	*R* ^2^	Model	*R*	*R* ^2^
1	0.606ª	0.367	0.365	6.09	0.367	203.93	0.000	
2	0.626b	0.392	0.389	5.98	0.026	14.82	0.000	1.926
3	0.640c	0.410	0.405	5.90	0.018	10.52	0.001	

a. Predictors Variables: (Constant), Family-school relationship; b. Predictors Variables: (Constant), Family-school relationship; Social Impact (negative); c. Predictors Variables: (Constant), Family-school relationship; Social Impact (negative); Leisure activities at home. d. Dependent Variable: Educational activities and performance at home.

The stepwise regression analysis yielded three models, of which the third offered the greatest explanatory power. The multiple correlation coefficient was *R* = 0.640 and the coefficient of determination *R*^2^ = 0.410 which was adjusted to *R*^2^ = 0.405. The third model showed that the independent variable with the most impact on the dependent variable was family-school relationship (β = 0.544), as was the case in the Early Childhood Education stage. Similarly, social impact (β = −0.147) and leisure activities at home (β = 0.139) explained the positive perception of academic performance at home during COVID-19 lockdowns (Table 5). In order to ascertain the validity of the model, the Durbin-Watson D statistic was calculated, which gave a value of *D* = 1.92 [according to Durbin and Watson (1951), a value close to 2.0 is considered valid].

In short, two different psychoeducational profiles were determined according to students’ stage of education. In Early Childhood Education, 35.5% of the variability in academic performance at home was explained by the quality of the family-school relationship and the emotional impact of lockdowns. However, in Primary Education, 40.5% was explained by the family-school relationship, the social impact, and the time and quality of leisure activities at home during COVID-19 lockdowns.

## 4 Discussion

This study sought to shed light on whether the impact of lockdowns on certain psychological and contextual variables gives rise to differential profiles in Early Childhood and Primary Education students. Similar correlations were found between the variables analyzed for both student groups, the most significant being that between academic performance at home and family-school relationship, followed by the correlation between emotional and social impact (Cachón-Zagalaz et al., 2020; Pozzoli et al., 2022). This trend was observed in both Early Childhood and Primary Education students, which highlights that despite the differences attributed to students’ stage of development, they show similar relationships in the contextual and psychological variables analyzed.

Related to the first hypothesis (H1), significant differences between Early Childhood and Primary Education students were observed in all the variables analyzed except for family-school relationship. This variable did not show a differential effect due possibly to the fact that communication between families and teachers was essential for the monitoring of online teaching and support when implementing home-schooling. However, families of Early Childhood Education students did indicate that they were more satisfied with the role of teachers (Cachón-Zagalaz et al., 2020; Tal et al., 2022).

Regarding effect size, the variable that showed a differential effect between the two stages was supervised leisure activities and collaboration at home. Younger students usually receive more one-on-one attention from adults and are supervised more. Consequently, the quality and frequency of interactions between parents and children is more conducive to children’s development by creating a motivating and stimulating learning environment (Oktaviana and Srianggita, 2021). Small effect sizes were shown in the other variables, whose direction showed greater resilience in Early Childhood Education students during lockdown (Giménez-Dasí et al., 2020; Egan et al., 2021).

In reference to correlations (hypothesis H2), the link between variables can be observed that show a similar direction in both stages. The most significant correlations were observed equally in both groups between family-school relationship and academic performance, followed by the correlation between social and emotional impact. It should be highlighted that the results are unsurprising given that the family-school relationship was key in the development of school activities outside the classroom, with teachers providing guidelines and support throughout the educational process (Pozzoli et al., 2022). The correlation between social and emotional impact in both stages was also expected given that although the variables are interdependent, they ultimately compose a single psychological construct.

In addition to those mentioned above, similar correlations were obtained between leisure activities and family-school relationship; co-responsibility and family-school relationship, and emotional impact and academic performance. The results are supported by evidence that the family-school relationship is an indicator of family involvement in the educational process, which is generalized in other informal educational contexts, resulting in higher academic achievement and cognitive stimulation (Oktaviana and Srianggita, 2021). Furthermore, co-responsibility in the family evidences a sense of shared responsibility for education in both formal and informal settings. Lastly, given that one’s emotional state influences cognitive performance, the fact that emotional impact was directly related to academic performance in both girls and boys who experienced lockdown was the expected result.

Related to the third hypothesis (H3), differential profiles in the evolutionary characteristics of Early Childhood and Primary Education students were shown between the two. The differences were characterized by the significance of the variables in relation to academic performance. The most significant differences found revealed that Early Childhood Education students exposed to the atypical circumstances and better coping mechanisms than Primary Education students were used.

The variables that were predictors of academic performance in Early Childhood Education students were family-school relationship and emotional impact. From a developmental perspective, it was expected that both variables would have a determining influence on the younger students. Regarding the family-school relationship, understood as the perception of the communication flow between teachers and families, and the support received to implement academic activities at home, it was also expected that both variables would have a decisive influence on the academic performance of the younger students (Tan et al., 2021).

In relation to the socioemotional impact of lockdown on schoolchildren, the variables social and emotional impact, taken independently, played a differential role in the results for academic performance at both stages. On the one hand, school experiences during the Primary Education stage contribute to the development of social skills due to the characteristics of group work and the relationships established in the classroom (Blair et al., 2018; Setiadi, 2020). Transferring school activities to the home environment inhibits the development of pro-social relationships that are cultivated in both curricular and play activities (Timmons et al., 2021). In contrast, emotional development in the Early Childhood Education stage focuses on the expression of affection toward family members which, in turn, leads to a positive attitude in relationships with others (Johnson et al., 2017). Many authors emphasize the importance of the role of parents and, more specifically, their behavior in relation to the emotional socialization of their children, which is significantly associated with behavioral problems in minors, especially in the case of negative emotions. This study has highlighted how emotional impact acts as a predictor of academic performance, based on the consequences of transferring teaching to the home, which entails greater parental involvement. The significance of the variable emotional impact on academic performance can be seen in the results of those children who had a higher score in the variable but lower academic performance. Owing to the fact that the family is paramount in the creation and development of a child’s first affective bonds and responsible for their security and protection (Guzmán et al., 2019; Orgilés et al., 2020), it could be expected that an atypical and problematic situation of change, such as COVID-19 lockdowns, would have a greater emotional impact on children who might cope less effectively with changes produced in the school environment.

The primary conclusions of the study highlight that socioemotional impact has a greater influence on Primary Education students in situations of lockdown. Situations that lead to deprivation of social development in habitual contexts could also be due to circumstances other than COVID-19, such as natural disasters (volcanic eruptions, hurricanes, etc.). Socioemotional wellbeing should not be limited to interaction with the family, given that peer experiences also contribute to the wellbeing and construction of children’s social identity (Balluerka-Lasa et al., 2020; Cheng, 2020; López-Bueno et al., 2020). Students aged 6–12 can benefit from the use of telematic tools that put them in communication with their peers and foster the social interaction needed to ensure socioemotional development even in atypical situations.

These findings highlight the role of parents in their children’s school performance. Beyond confinement situations, it has been shown how the involvement of parents in school activities improves both their expectations and their satisfaction (Sánchez and Dávila, 2022). Likewise, the improvement in performance and the evolutionary differences require of different support needs at each stage. Likewise, it is the family’s responsibility to provide a balance between shared leisure moments and school activities for a good socio-affective adaptation in confinement.

The results of the study lead to the conclusion that Early Childhood Education students, compared to Primary Education students, were less affected by the situation of lockdown in the factors studied. This could be due to the fact that at this early stage, children acquire their first learning experience in the family, which is the foremost context for their development and upbringing. They benefit from closer interaction between their family and their school, which fosters more meaningful learning.

Among the limitations of the study, it is found that the instrument did not allow student’s experience information to be collected directly. Due to the evolutionary and cognitive characteristics of the students, a hetero-informed questionnaire was used. In addition, an overrepresentation of female informants was found since mothers are more involved in reconciling work and family (Fernández-Freire et al., 2019).

Likewise, it would be advisable to evaluate other influential factors in socio-emotional development, such as self-concept. This information can guide the implementation of strategies that contribute to the comprehensive development of the student.

## Data availability statement

The raw data supporting the conclusions of this article will be made available by the authors, without undue reservation.

## Ethics statement

The studies involving humans were approved by Comité ético de la Universidad de Córdoba. The studies were conducted in accordance with the local legislation and institutional requirements. The participants provided their written informed consent to participate in this study. Written informed consent was obtained from the individual(s) for the publication of any potentially identifiable images or data included in this article.

## Author contributions

NS-D: Conceptualization, Funding acquisition, Writing – original draft, Writing – review & editing. EA: Conceptualization, Data Curation, Funding acquisition, Formal Analysis, Writing – original draft, Writing – review & editing. RM-S: Funding acquisition, Writing – review & editing.
